# Improved biodegradation of hydrophobic volatile organic compounds from the air stream in a multilayer biofilter

**DOI:** 10.1016/j.mex.2019.09.020

**Published:** 2019-09-18

**Authors:** Mehdi Mokhtari, Yaghoub Hajizadeh, Negar Jafari, Ali Asghar Ebrahimi, Ali Abdolahnejad

**Affiliations:** a Environmental Science and Technology Research Center, Department of Environmental Health Engineering, Shahid Sadoughi University of Medical Sciences, Yazd, Iran; b Department of Environmental Health Engineering, Faculty of Health, Environmental Research Center, Research Institute for Primordial Prevention of Non-Communicable Disease, Isfahan University of Medical Sciences, Isfahan, Iran

**Keywords:** Improved biodegradation with a multilayer biofilter, Biodegradation, Rhamnolipid, Biofiltration, VOCs

## Abstract

Biofiltration of n-hexane as a representative of hydrophobic volatile organic compounds (VOCs) at presence and absence of *rhamnolipid* biosurfactant was studied using a multilayer biofilter packed with scoria, compost, poplar tree skin and sugar beet pulp, for 131 days. The concentration of n-hexane was measured by a gas chromatograph coupled with a flame ionization detector (GC/FID). The results showed that the mean removal efficiency (RE) of n-hexane at the presence of the biosurfactant was two times higher than that at absence of the biosurfactant. According to the results, *rhamnolipid* can enhance the efficiency of biofiltration of VOCs from polluted air streams.

## Graphical abstract

**Figure:**
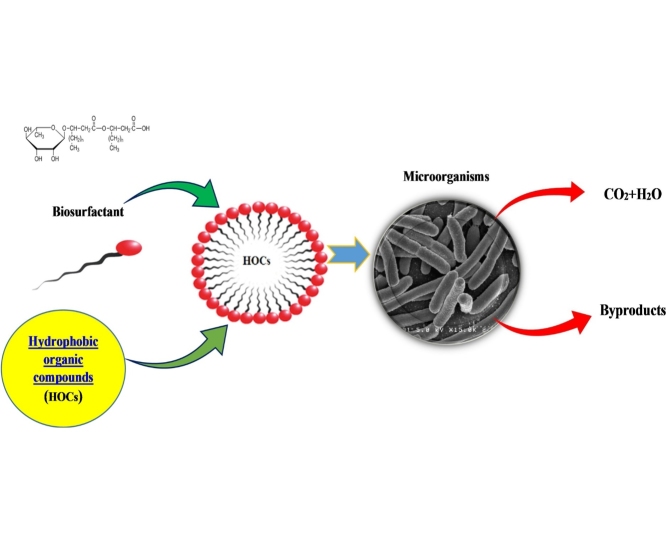

**Specification Table**

**Table:** 

**Subject Area**:	Environmental Science
**More specific subject area**:	Air pollution control
**Protocol name**:	Improved biodegradation with multilayer biofilter
**Reagents/tools**:	GC/FID, model: Varian CP-3800, USA, column characteristics: type: CP-sil5 CB, column: 15.0 m × 0.25 mm × 0.45 μm, injector characteristics: the injector temperature was constant at 200 °C, carrier gas: Nitrogen (N_2_) at a flow rate of 1.2 ml min^−1^, make-up gas: nitrogen (N_2_) at a flow-rate of 25 ml min^−1^.
**Experimental design**:	All sampling and parameters analysis were measured according to in our previous study [1].
**Trial registration**:	Not required
**Ethics**:	Not required

**Value of the Protocol**

**Table:** 

• Biofiltration of VOCs is only dependent on its own gas solubility and bioavailability [2,3]. • Thus, these pollutants are usually poorly removed by biofiltration [4,5,6]. • The application of a chemical or biological surfactant can be one of the practical options to enhance the bioavailability and solubility of hydrophobic organic compounds (HOCs) [7,8,9,10]. • Rhamnolipids are anionic bio-surfactants which are produced by Pseudomonas sp. Bacteria. Biosurfactants compare to chemical surfactants are more effective, less toxic, environmentally friendly and more biodegradable. It increases the water solubility of HOCs and thereby facilitates the biodegradation of these compounds [11,12,13,14].

## Description of protocol

### Biofilter set-up and operation

**Fig. 1:**
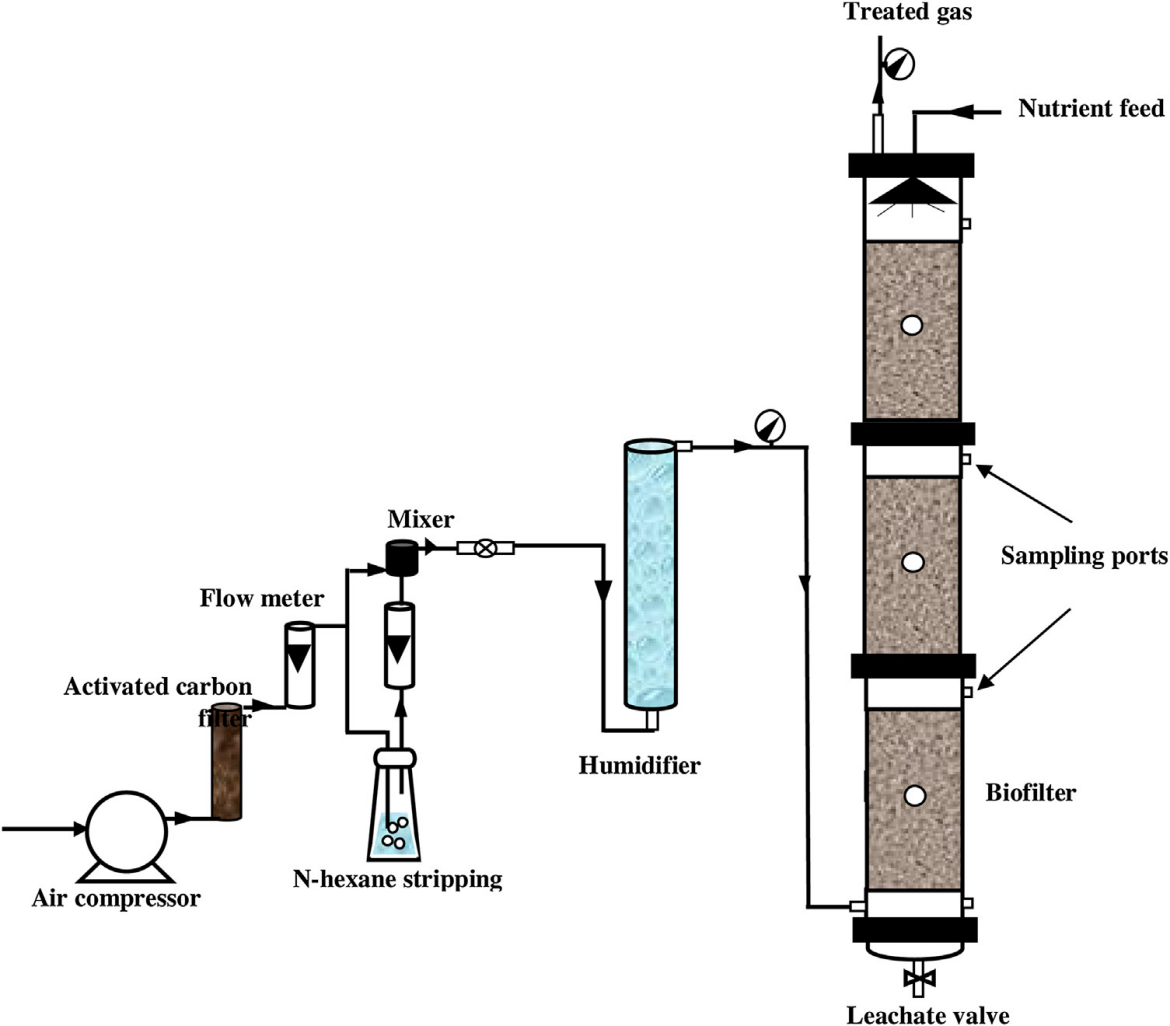
Schematic of the laboratory scale biofilter.

The biofilter was included a stainless steel column with 120 cm height, 15 cm inner diameter and effective volume of15.8 L (three sections, each 40 cm height (Fig. 1). Airflow was supplied by a 150 L compressor. Four different mixtures of materials consisted of 40% compost, 20% scoria, 20% sugar beet pulp, and 20% poplar tree skin were used as packing materials in the biofilter. More details are available in our previous study [1]. After the start-up phase, the performance of biofilter in various operational conditions at presence and absence of the biosurfactant was surveyed during the operation period (131 days in 6 phases). Operating conditions of each phase are presented in Table 1. To decrease the adaptation time of microorganisms in the biofilter bed, fresh activated sludge of the wastewater treatment plant was used as an inoculum source for the biofilter bed.

**Table 1:** Summary of operational conditions of the biofilter.

Phase of operation	EBRT (s)	Biosurfactant addition	Inlet concentration range (gm^−3^)	Inlet loading range (gm^−3^ h^-1^)	Operation time (day)	Sample size (Number)*
*Start-up*	120	NO	0.18–0.41	6.2–14.08	31	62
*I*	120	NO	0.19 - 0.98	6.7–33.4	25	50
*II*	60	NO	0.21–1.01	14.25–68.5	23	46
*III*	30	NO	0.18–1.0	25.7–137.1	23	46
*IV*	120	YES	0.24–0.86	8.4–29.3	20	40
*V*	60	YES	0.18–1.03	12.91–70.6	20	40
*VI*	30	YES	0.19–0.99	27.03–135.34	20	40

* Samples were taken and analyzed in duplicate.

### Chemicals

N-hexane and all the other chemicals for preparation of the nutrient solution were obtained from *Merck Company* (with a purity 99%). The composition of the nutrient solution (per liter of distilled water) was as follow: 0.5 g NaCl, 0.1 g NaHCO_3_ (for control of pH in the liquid medium), 0.15 g KH_2_PO4, 0.3 g MgSO_4_, 0.01 g FeSO_4_, 0.5 g NH_3_SO_4_,1.9 g ClNH_4_, 0.03 g MnSO_4_, 0.03 g ZnSO_4_ [1,15]. *Rhamnolipid* biosurfactant with a purity of 95.0% was obtained from *Institute of chemical engineering of Iran* (ICEI). The concentrations of 50, 100, 200 and 300 mg/l of the biosurfactant were prepared in nutrient solution and individually introduced to the biofilter in optimum EBRT (120 s). After application, 300 mg/l was selected as an optimal concentration of *rhamnolipid* and used in the rest of the experiments.

### Analytical methods

Concentrations of n-hexane in inlet and outlet of the biofilter were analyzed using a gas chromatograph equipped with an FID (GC/FID*, Varian CP-3800, USA*) with column details of 15.0 m ×0.25 mm ×0.45 μm (CP-sil5 CB). The temperature of the detector was set to 280 °C. Nitrogen (N_2_) at a flow rate of 1.2 and 25 ml min^−1^ was used as a carrier and make-up gas, respectively. The injector temperature was constant at 200 °C. More details are available in our previous study [1]. For draw up of the calibration curves, various concentrations of n-hexane were injected into glass bottles sealed with a Teflon and analyzed after stabilization. For measurement of n-hexane concentrations, any time 0.5 ml of gas was injected into the GC-FID with a gas-tight syringe (*Hamilton, USA*). Total air samples taken from inlet and outlet of the biofilter during the operation period (131 days; without the start-up period) was 262. To evaluate the removal efficiency of n-hexane in the biofilter, two samples were taken daily and the mean values of them were used for the calculation of removal efficiency and elimination capacity.

### Effect of rhamnolipid biosurfactant on the performance of the biofilter

**Fig. 2:**
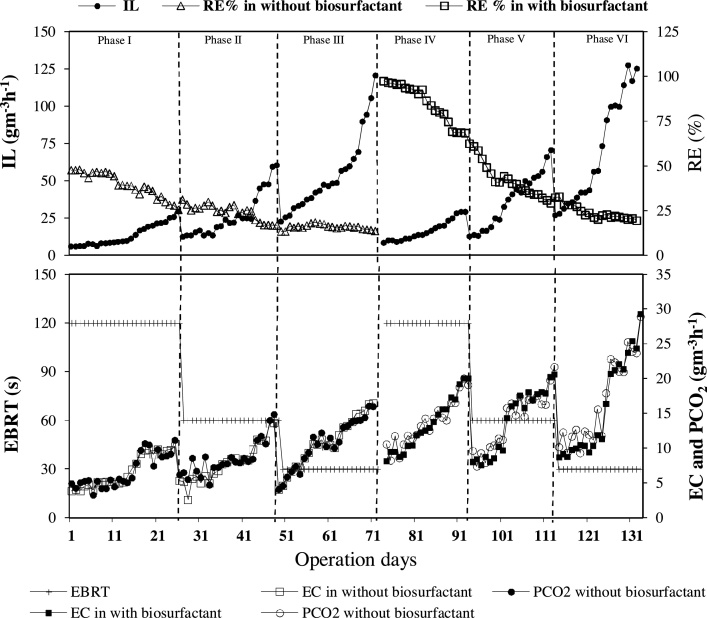
Influence of rhamnolipid biosurfactant on n-hexane removal efficiency, elimination capacity and CO_2_ production.

The trend of removal efficiency (RE) and elimination capacity (EC) of n-hexane with and without the biosurfactant in different inlet loading rate (IL) was illustrated in Fig. 2. The removal efficiency of n-hexane in the biofilter without addition of *rhamnolipid* was 46.8%, whereas, with the addition of *rhamnolipid*, the removal efficiency was increased. So that, in concentrations of 50, 100, 200 and 300 mg/l *rhamnolipid*, the removal efficiency reached to 47.26%, 56.76%, 73.24% and 85.13%, respectively. There is a positive linear relationship between the solubility of HOCs and the biosurfactant concentrations. Therefore, with increasing the concentration of the biosurfactant the removal percentage of n-hexane was increased [1,16].

### Conclusions

Single biofilter due to low water solubility and low bioavailability of HOCs has not high removal efficiency for n-hexane. Our results confirmed that introduction of rhamnolipid as a proper biosurfactant to the biofilter media with favorable concentration can notably increase the removal efficiency of HOCs from the air stream. So that, in our study, the mean removal efficiency of n-hexane by adding *rhamnolipid* biosurfactant to the biofilter media and under optimum EBRT (120 s) was increased from 46.8% to 85.13%. Because *rhamnolipid* biosurfactant by reducing surface tension and causing micelles formation can increase the water solubility and bioavailability of HOCs for microorganisms in the biofilter media.

## Declaration of Competing Interest

The authors of this article declare that there is no conflict of interests.
